# Immersion in Raloxifene does not significantly improve bone toughness or screw pull-out strength in multiple in vitro models

**DOI:** 10.1186/s12891-021-04342-1

**Published:** 2021-05-22

**Authors:** Michael R. Eby, Danielle M. Cristino, Matthew Counihan, Kendall M. Masada, Jaimo Ahn, Michael W. Hast

**Affiliations:** 1 Department of Orthopaedic Surgery, University of Pennsylvania, Philadelphia, PA USA; 2 Biedermann Lab for Orthopaedic Research, University of Pennsylvania, 3450 Hamilton Walk, 373A Stemmler Hall, Philadelphia, PA 19104 USA; 3 Department of Orthopaedic Surgery, University of Michigan, Ann Arbor, MI USA

**Keywords:** Raloxifene, Fracture repair, Proximal humerus, Cadaveric, Biomechanics

## Abstract

**Background:**

Failure of surgical fixation in orthopaedic fractures occurs at a significantly higher rate in osteoporotic patients due to weakened osteoporotic bone. A therapy to acutely improve the mechanical properties of bone during fracture repair would have profound clinical impact. A previous study has demonstrated an increase in mechanical properties of acellular cortical canine bone after immersion in raloxifene. The goal of this study was to determine if similar treatment yields the same results in cancellous fetal bovine bone and whether this translates into a difference in screw pull-out strength in human cadaveric tissue.

**Methods:**

Cancellous bone from fetal bovine distal femora underwent quasi-static four-point bending tests after being immersed in either raloxifene (20 μM) or phosphate-buffered saline as a control for 7 days (*n* = 10). Separately, 5 matched pairs of human osteoporotic cadaveric humeral heads underwent the same procedure. Five 3.5 mm unicortical cancellous screws were then inserted at standard surgical fixation locations to a depth of 30 mm and quasi-static screw pull-out tests were performed.

**Results:**

In the four-point bending tests, there were no significant differences between the raloxifene and control groups for any of the mechanical properties - including stiffness (*p* = 0.333) and toughness (*p* = 0.546). In the screw pull-out tests, the raloxifene soaked samples and control samples had pullout strengths of 122 ± 74.3 N and 89.5 ± 63.8 N, respectively.

**Conclusions:**

Results from this study indicate that cancellous fetal bovine samples did not demonstrate an increase in toughness with raloxifene treatment, which is in contrast to previously published data that studied canine cortical bone. In vivo experiments are likely required to determine whether raloxifene will improve implant fixation.

## Background

Osteoporotic fractures accounted for an estimated 9 million new fractures worldwide in the year 2000 and more than 2 million fractures in the United States in 2005 [1, 2]. Globally, it is estimated frailty has a 10.7% prevalence in populations over the age of 65, which translates to an estimated 10.2 million Americans whom suffered from the disease in 2010 [3, 4]. These numbers are projected to continue increasing in prevalence as our society ages and average lifespan lengthens. In the United States alone, projections estimate upwards of 275,000 proximal humerus fractures by 2030 [5]. Osteoporotic hip fractures are expected to reach 6.3 million worldwide by the year 2050 [1]. The total population facility-related hospital cost of osteoporotic fractures in the United States from the year 2000–2011 averaged $5.1 billion per year, higher than the respective facility-related costs of myocardial infarction ($4.3 billion) or stroke ($3.0 billion) [6]. In addition to these costs, we must also consider morbidity from loss of function, mortality from associated complications, and the socioeconomic impact of lost work productivity and strain on caretakers among other downstream effects [7].

**Fig. 1:**
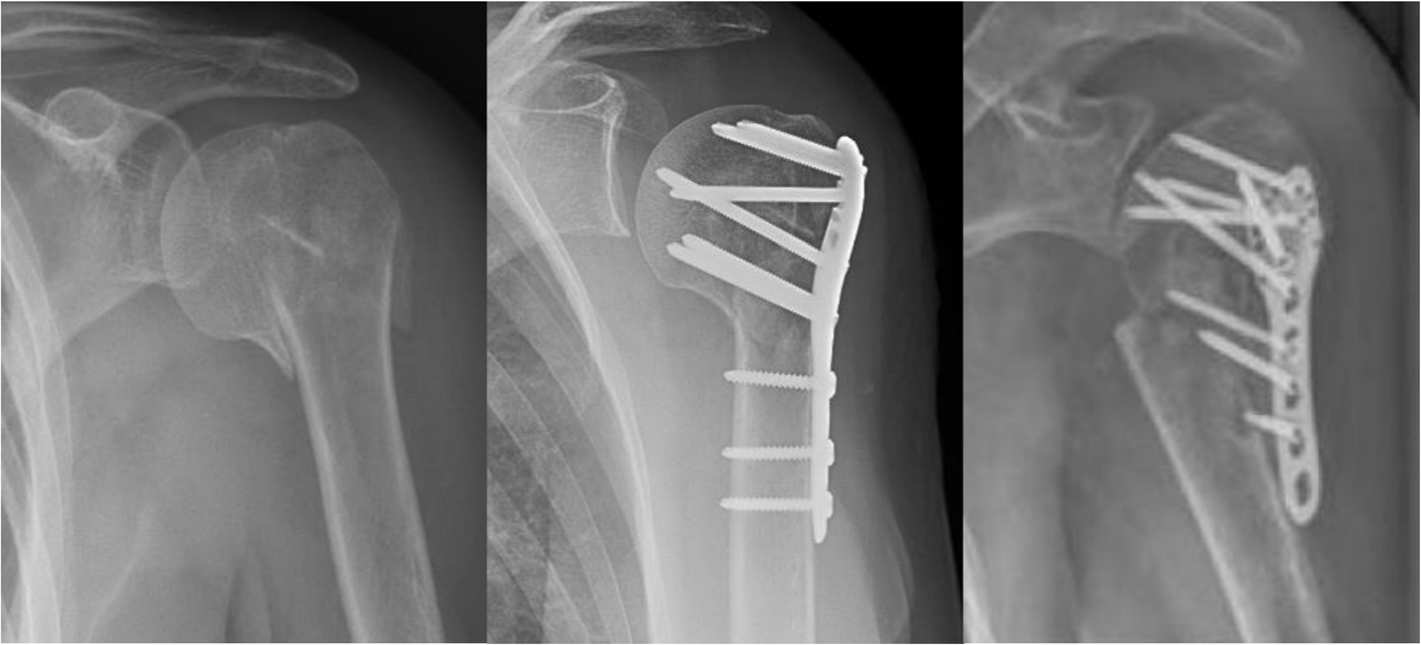
Proximal humerus fractures (**a**) preoperatively (**b**) status post open reduction, internal fixation (**c**) status post failure of fixation

Many of the challenges regarding successful reconstruction and repair of fractures in the elderly are associated with the decreased mechanical properties of osteoporotic bone. Complications are common and include implant failure, malunion, nonunion, collapse and screw cut out. (Fig. 1) One study demonstrated that proximal humerus fracture reconstructions using internal fixation strategies had a 49% complication rate [8]. This included malunion, implant pull-out, and primary and secondary screw perforation of the shoulder joint. Similarly, a systematic review of osteoporotic hip fractures treated by all types of fixation found a failure rate of 14.8% in nondisplaced femoral neck fractures and 41% in displaced femoral neck fractures [9]. A separate implant-specific study showed failure rates greater than 50% in osteoporotic hip fractures [10]. Structural augments, such as allografts [11, 12] and bone substitute materials [13, 14], are being utilized more frequently, but their long-term utility is not well understood and their complication rates remain unacceptably high [15]. It remains difficult to estimate the degree to which poor clinical outcomes of fracture reconstructions influences the decision between fixation and joint arthroplasty in articular fractures of the shoulder, hip, elbow, and knee.

Improvement of the implant-bone interface is central to the advancement of osteoporotic fracture care by reducing the incidence of screw loosening or pull-out. Implant advances such as intramedullary nailing, locked plating, variable angle screws and pegs have all contributed to decreased failure of constructs in osteoporotic bone by tailoring their design to weaker bone [16]. The majority of research has focused on the design of implant constructs, while relatively little attention has been paid to the fundamental source of the problem: the decreased mechanical properties of osteoporotic bone. A longstanding goal in orthopaedics remains to identify a therapeutic agent to acutely improve the mechanical quality of the weaker bone for fracture fixation. This agent would be administered at the time of fixation and act rapidly enough to improve the bone as it heals, preventing implant failure. Several medications, including bisphosphonates and teriparatide, have successfully increased bone mineral density in osteoporotic bone [17–19]. However, orthopaedic surgeons use these drugs to prevent additional fragility fractures, so their role in acute fracture care is less clear. The use of bisphosphonates for acute fracture care is controversial, with some suggesting it delays fracture healing [20]. Others have found that bisphosphonate therapy has no appreciable effect on fracture healing [21, 22] and may even negatively influence fracture healing [23, 24] A meta-analysis of 380 patients across 5 randomized controlled trials of teriparatide in acute fracture care showed no significant improvements in fracture healing rates or time to radiographic healing [25].

Raloxifene is a selective estrogen receptor modulator that is previously known to improve bone quality when taken systemically over a prolonged period of time [26]. Its primary mechanism of action involves modification of gene expression, as well as a decrease in the quantity and activity of osteoclasts [27]. However, it appears that raloxifene’s effects are not entirely explained by this mechanism. A recent in vitro study of raloxifene investigated a potential secondary mechanism for improving bone quality [28]. Interestingly, cortical canine bones immersed in raloxifene showed improved toughness, which challenges the assumption that a cellular response is required to improve bone properties with raloxifene. From an engineering standpoint, measurements such as toughness, energy to fracture, and post-yield energy are indicators of a material’s ability to deform and absorb energy prior to fracturing.

It is unknown whether this potential acellular effect translates to cancellous bone and is substantial enough to produce a clinically relevant improvements in bone toughness and screw pull-out strength, both of which play an important role in fragility fracture repair. The purpose of this study was twofold: First, to attempt to extend the findings of Gallant et al. to cancellous bone in an in vitro animal model representative of osteoporosis (fetal bovine femora); and second, to test the utility of raloxifene application with human cadaveric tissue in a clinically relevant application (proximal humerus fracture repair). We hypothesized that raloxifene immersion of fetal bovine bone would increase toughness and improve screw pull-out resistance in human cadaveric specimens.

## Methods

This study included deidentified cadaveric human and bovine specimens. All institutional guidelines regarding the use of these specimens were strictly followed. Approvals from the Institutional Animal Care and Use Committee (IACUC) and the Institutional Review Board (IRB) were not required by our institution for this study. Donors provided informed consent for use of their bodies for medical research (Source: ScienceCare, Phoenix, AZ, USA).

The first portion of this study involved four-point bending tests of cancellous bone beams harvested from the distal femora of fetal bovine specimens, similar to a previously published study [29]. Cancellous bone was tested because it is representative of bone in the head of the humerus and fetal bovine specimens were selected because they represent a reasonable model of the osteoporotic condition [29]. Ten specimens were acquired for this study. Using a reciprocating saw with a 1 mm blade, a total of 20 specimens were harvested from the distal metaphysis of the femora (~ 33 weeks gestation). The sample size of *n* = 10 was based on a power analysis of toughness results from Gallant et al. [28], which yielded a 0.988 power value. Individual specimens were carefully hand sanded into 25 × 4 × 1.5 mm (+/− 0.05 mm) blocks and digital calipers were used to verify dimensions. The specimens were thoroughly cleaned of soft tissue, sonicated for 30 s at room temperature, and frozen until day 1 of the soak (− 20 °C). Specimens were submerged in one of two solutions in a sterile container: control (600 μL of dimethylsulfoxide (DMSO), 1% penicillin-streptomycin and phosphate-buffered saline (PBS)) or raloxifene (20 μM of raloxifene suspended in 600 μL of DMSO and 1% penicillin-streptomycin in PBS). DMSO was constant at 0.04% vol/vol. The bone beams and solutions were placed on a plate shaker for 7 consecutive days and held at 4 °C.

**Fig. 2:**
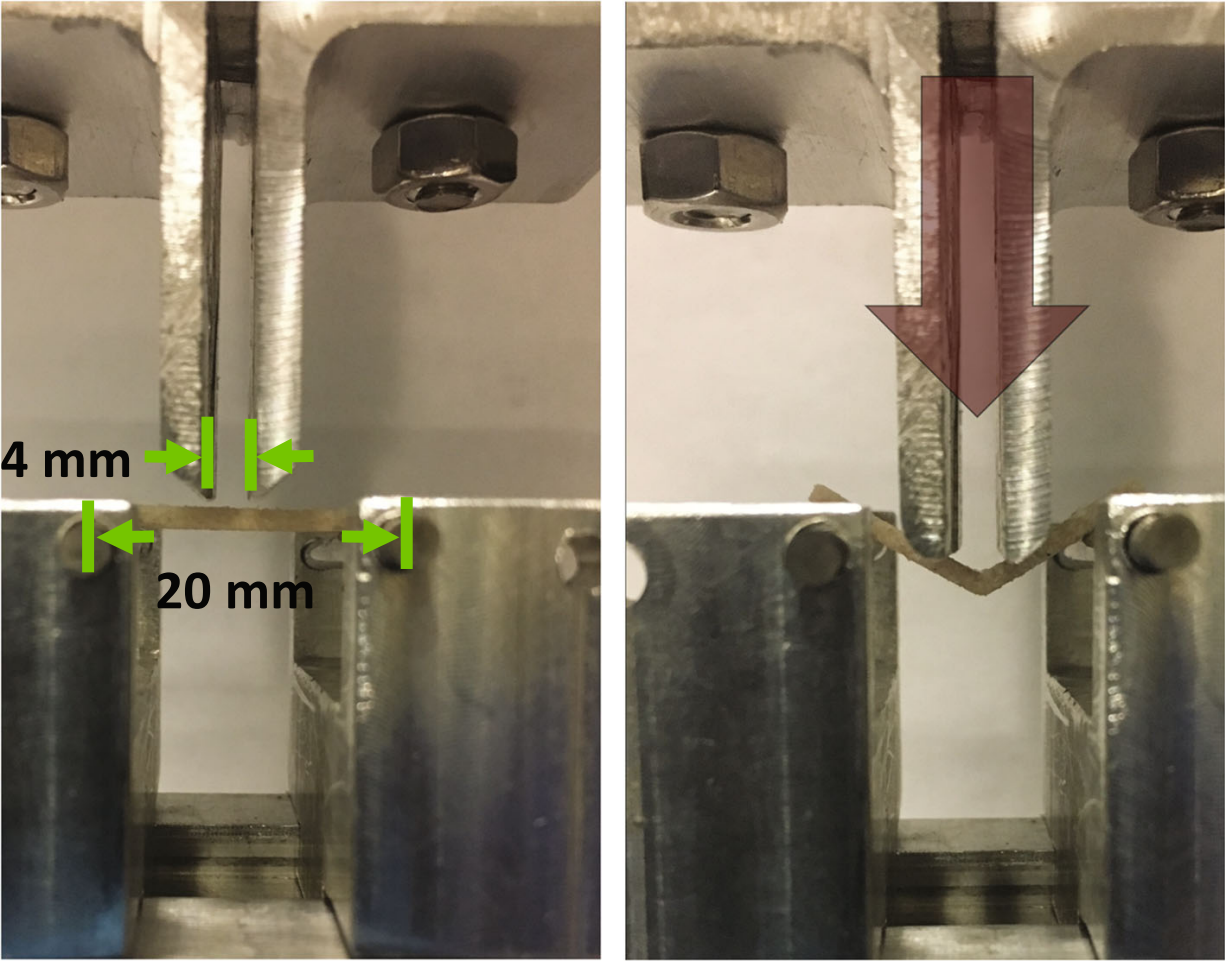
Photographs of the 4-point bending test setup. The custom jig was constructed of aluminum with stainless steel rollers. A universal joint was installed above the top anvils to ensure that 4 points of contact could be maintained at all times throughout testing

Specimens were subjected to a four-point bending protocol, adapted from ASTM standard C1161–18. (ASTM International, West Conshohocken, PA) All specimens were brought to room temperature prior to testing and remained hydrated until being placed on a universal test frame (Instron 5542; Norwood, MA) equipped with a 50 N load cell and a custom jig (Fig. 2). The beams were quasi-statically loaded to failure at a rate of 0.1 mm/sec. The following mechanical properties were calculated: stiffness (N/mm), ultimate force (N), bending rigidity (Nm^2^), modulus of elasticity (MPa), ultimate stress (MPa), toughness (J/m^3^), energy to fracture (J/m^3^), and post-yield energy to failure (J/m^3^). We were particularly interested in the stiffness and energy results (toughness, energy to fracture, energy post-yield) because they are representative of bone quality, brittleness and fracture resistance, which relate to failures of frailty fracture reconstructions.

**Fig. 3:**
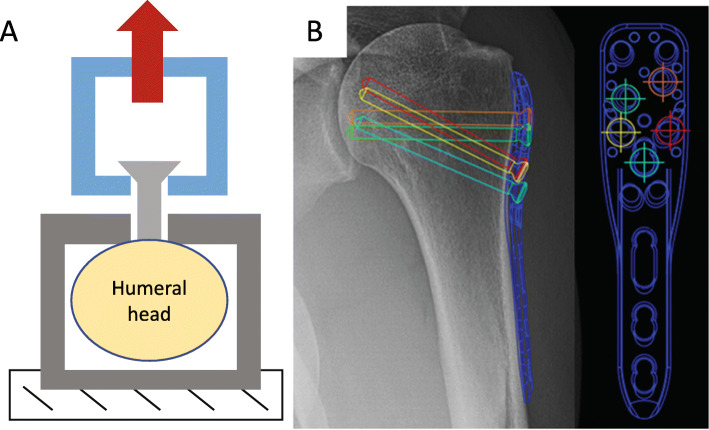
**a** A schematic diagram of the screw pull-out test (not to scale). The humeral head was placed in an aluminum box with a hole that was larger than the screw head. To create orthogonal pulling, screw heads were captured in a custom aluminum jig that was connected to a universal joint, which was in line with the load cell and actuator (not shown). **b** Locations of screw placement for pull-out testing for locations 1–5. Note: plate and drill tower were utilized for consistent trajectory but removed prior to screw insertion and pull-out testing

The second portion of this study utilized 5 matched pairs (right and left) of cadaveric proximal humeri from donors that were confirmed osteoporotic by dual energy X-ray absorptiometry (DEXA) scan. (4 female, 1 male, average age 81.8 years, age range 74–87 years, average T-score of the most osteoporotic vertebral body − 3.02) Specimens were skeletonized with standard gross dissection techniques and the proximal humeri were isolated from the rest of the shoulder joint with an oscillating saw. With the exception of the lateral wall cortex (the cortex the screws were placed in), the humerii were decorticated with an oscillating saw blade to allow for permeation of cancellous bone during soaking. Pilot holes for 5 screws were drilled into each specimen with a 2.5 mm drill bit (Trajectories demonstrated in Fig. 3) using a LCP Proximal Humerus locking plate and corresponding drill guide (DePuy Synthes, West Chester, PA). Prepared specimens were soaked in solution after drilling to allow for permeation into the bone-screw interface. Each matched pair had one randomized side soaked in 20 μM raloxifene solution and the contralateral side soaked in control solution for 1 week, following the same protocol as the first portion of the study.

Screw pull-out testing was performed in accordance with ASTM F543–17 (ASTM International, West Conshohocken, PA). Testing was performed at the time of soak completion after being brought to room temperature. Hydration was maintained before and after individual screw placement and testing, which took less than 5 min to perform. A 3.5 mm cortical screw (DePuy Synthes, Warsaw, IN) was inserted unicortically (without the plate) into each of the randomly selected 5 pre-drilled trajectories (Fig. 3a) to a depth of 30 mm. The screw was removed quasi-statically at a rate of 0.03 mm/sec on a universal testing frame (TA Electro-Force 3550; Eden Prairie, Minnesota) equipped with a 1110 N load cell, utilizing a standard screw pullout setup that captures the screw head and applies a load coincident with the long axis of the screw (Fig. 3b). Recorded force-displacement data collected at 100 Hz was used to determine ultimate pull-out force.

Statistical analysis was performed in SigmaStat 4.0 (Systat Software, San Jose, CA). For all analyses, Shapiro-Wilk tests were used to test for normality. For the four-point bend tests, all the data were normal, so two-tailed, two-sample, equal variance Student t-tests were used to assess unknown responses across groups. For the screw pull-out study, a two-way analysis of variance was performed. Manipulation was one main effect (Raloxifene versus control), location of the screw was the other main effect (5 locations), and donor was a random effect. Because data did not pass the Shapiro-Wilk tests for normality, least square means were calculated and pairwise multiple comparison procedures were performed with the Holm-Sidak method. The significance level was set at α = 0.05 for all tests.

## Results

**Fig. 4:**
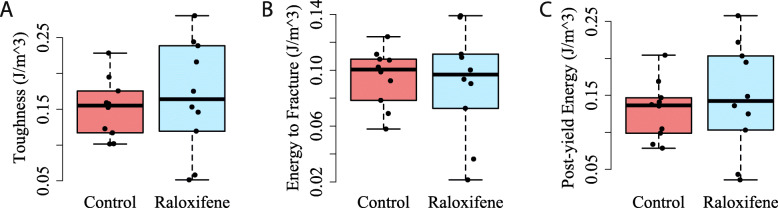
Box and whisker plots of (**a**) toughness, (**b**) energy to fracture, and (**c**) post-yield energy during 4-point bends. There were no significant differences between control and raloxifene immersed samples for any of these measures

In the fetal bovine experiment, there were no significant differences between the raloxifene and control groups for any of the mechanical properties determined from four-point bending. The stiffness values for the control and raloxifene groups were 1.7 ± 0.7 N/mm and 1.4 ± 0.6 N/mm, respectively (*p* = 0.333). The toughness values were 0.15 ± 0.04 J/m^3^ and 0.17 ± 0.08 J/m^3^ (*p* = 0.615), the energy to fracture values were 0.09 ± 0.02 J/m^3^ and 0.09 ± 0.04 J/m^3^ (*p* = 0.794), and the post-yield energy values were 0.13 ± 0.04 J/m^3^ and 0.15 ± 0.07 J/m^3^ (*p* = 0.531), for the control and raloxifene groups, respectively (Fig. 4). Other comparisons of mechanical properties did not show significant differences: ultimate force (*p* = 0.560), bending rigidity (*p* = 0.422), modulus of elasticity (*p* = 0.303), and ultimate stress (*p* = 0.569) (Table 1).

**Table 1:** Mechanical Properties derived from four-point bending of Control group and Raloxifene treatment group (metaphyseal fetal bovine femora)

Mechanical Property	Control (***n*** = 10)	Treatment (***n*** = 10)	***P***-Value
**Stiffness (N/mm)**	1.7 ± 0.7	1.4 ± 0.6	0.333
**Ultimate Force (N)**	1.69 ± 0.423	1.56 ± 0.527	0.560
**Bending Rigidity (Nm**^**2**^**)**	0.0004 ± 0.0002	0.00038 ± 0.000162	0.422
**Modulus (MPa)**	226 ± 86.5	190 ± 62.2	0.303
**Ultimate Stress (MPa)**	4.05 ± 1.08	3.77 ± 1.08	0.569
**Toughness (J/m**^**3**^**)**	0.15 ± 0.04	0.17 ± 0.08	0.615
**Energy to Fracture (J/m**^**3**^**)**	0.09 ± 0.02	0.09 ± 0.04	0.794
**Post-yield Energy (J/m**^**3**^**)**	0.13 ± 0.04	0.15 ± 0.07	0.531

**Fig. 5:**
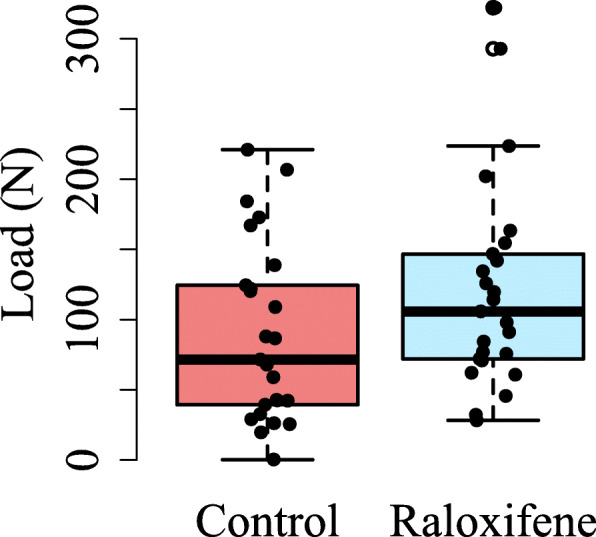
Box and whisker plot of pull-out forces for all samples. Although the samples immersed in raloxifene had a slightly higher mean value, there was no statistical significance between groups

For the cadaveric tests, ultimate screw pull-out loads were 89.5 ± 63.8 N and 122 ± 74.3 N for the pooled control and raloxifene groups, where each screw was treated as a unique data point (Fig. 5). There were no significant differences found due to changes in screw location within the control or Raloxifene groups (Tables 2 and 3). When analyzing changes in treatment within locations of the screws, only location 4 was found to have a significant difference (*p* = 0.018).

**Table 2:** Summary of two-way analysis of variance for the screw pull-out testing. All units are Newtons

Comparisons for factor: Treatment within Location		
Comparison	Diff of Means relative to control (N)	P
Ralox vs. Control (Location 1)	45.50	0.276
Ralox vs. Control (Location 2)	15.36	0.711
Ralox vs. Control (Location 3)	−24.48	0.556
Ralox vs. Control (Location 4)	102.06	0.018
Ralox vs. Control (Location 5)	22.66	0.586

**Table:** **Table 3** Pairwise comparisons for screw locations within Control and Raloxifene groups. All units are Newtons

Comparisons for factor: Screw Location within Control			Comparisons for factor: Screw Location within Ralox		
Comparison	Diff of Means (N)	P	Comparison	Diff of Means (N)	P
1 vs. 2	44.94	0.863	1 vs. 2	75.08	0.425
1 vs. 3	26.48	0.949	1 vs. 3	43.50	0.829
1 vs. 4	39.56	0.878	1 vs. 4	17.00	0.899
1 vs. 5	54.24	0.782	1 vs. 5	77.08	0.435
2 vs. 3	71.42	0.576	2 vs. 3	31.58	0.832
2 vs. 4	5.38	0.897	2 vs. 4	92.08	0.248
2 vs. 5	9.30	0.969	2 vs. 5	2.00	0.962
3 vs. 4	66.04	0.630	3 vs. 4	60.50	0.623
3 vs. 5	80.72	0.445	3 vs. 5	33.58	0.887
4 vs. 5	14.68	0.979	4 vs. 5	94.08	0.246

## Discussion

The high rate of fixation failure in osteoporotic fractures poses a significant challenge to surgeons and their patients, warranting exploration of novel approaches to improving fixation strength. Few treatments have shown promise in addressing the underlying issue of poor bone quality within the timeframe of fracture healing. We determined that immersion in raloxifene did not improve bone material properties in fetal bovine bone and screw pull-out strength in osteoporotic human cadaveric bone. Unlike the results of previous research [28], toughness in 4-point bending was not increased by soaking the specimens in raloxifene. A trend towards a higher screw pull-out strength in osteoporotic cadaveric bone was observed for bone treated with raloxifene, however the results were not statistically significant.

Raloxifene is a commonly used drug in the treatment of osteoporosis, but the traditional belief was that it required a cellular response to improve bone density and that this response took longer than fracture healing timeframes [27]. Based on this premise, orthopaedic surgeons typically begin treatment with raloxifene or similar medications to prevent additional osteoporotic fractures (instead of treating the current fracture acutely). To the authors’ best knowledge, this is the first investigation to determine whether an acellular effect of raloxifene is significant enough to produce a clinically relevant improvement in acute osteoporotic fracture fixation.

This experiment only examined the in vitro response of tissue immersed in a raloxifene solution, and an in vivo animal model would provide a clearer understanding of the biological mechanisms at play. For example, we could measure the cellular uptake locally within a fracture as well as gauge the cytotoxicity of local administration of raloxifene. The classical mechanism of action is based on the general premise that it must enter the nucleus and bind to an estrogen receptor to effect changes in gene expression. Because it is a selective estrogen receptor modulator, raloxifene does not share all effects with estrogen. While estrogen has a myriad of effects depending on the type of tissue it is exposed to [27], the full mechanism of action of raloxifene is still uncertain. Further, raloxifene has been shown to decrease the number and activity level of osteoclasts [30]. These cell-mediated mechanisms explain the clinical effect of prolonged raloxifene treatment systemically, in which bone mineral density is increased.

Recent success with an in vitro model suggests that acellular mechanisms may influence the mechanical properties of bone. Gallant et al. suggested that hydroxyl groups on the molecular structure of raloxifene may be the key to a short-term, acellular effect of raloxifene on the mechanical properties of bone – these groups affect the interface between mineral bone and collagen and may increase hydration of the bone [28]. Multiple studies have demonstrated improved mechanical properties of bone that is more hydrated [31, 32]. This potential mechanism would allow for a much faster effect than the steroid based effect on gene expression. This would theoretically allow raloxifene to be utilized locally in an acute perioperative setting to lower the likelihood of implant failure by acutely increasing mechanical quality of osteoporotic bone. It remains unclear why an increase in toughness of acellular bone was observed after exposing cortical canine bones to raloxifene [28], and ex vivo soaking of human femora in raloxifene enhanced post-yield behavior at the structural level when measured with reference point indentation techniques [33]. We did not find similar results in the current experiment, where there were no significant differences in immersed cancellous bones of fetal bovines and osteopenic human cadavers. This area of research deserves future consideration and experimentation.

There were several differences between this study and the previous study by Gallant et al. The current study tested cancellous bone from fetal bovine femora and human cadaveric humeri, whereas the previous study was performed with cortical bone harvested from adult canine femora and adult human tibiae. In comparison to the study by Gallant et al., we increased the concentration of raloxifene tenfold (20 μM vs. 2 μM). Although it was posited that increasing the concentration would have a stronger effect on outcome, preliminary testing did not demonstrate a positive or negative concentration-dependent relationship to mechanical properties (pilot data not shown). We utilized a shorter soaking time (7 days vs. 14 days), however the authors of the previous study note that they achieved similar results with a 2 day soak as they did with a 14 day soak, which gives us confidence that 7 days is a reasonable soak duration. Finally, we performed our soaks at a lower temperature after being unsuccessful at maintaining sterility for 14 days at 37 degrees C.

Fetal bovine tissue provided a reasonable model for osteoporotic bone. It has been demonstrated that fetal bovine bone tissue has decreased elastic modulus and yield stress [29] than intact adult cortical bovine or human bone [34–37]. Additionally, the porosity of fetal bone is higher than that reported for healthy adult cortical bone which is in the range of 5 to 12% [29, 36, 37]. Additionally, it is believed that the biologic composition of the fetal bone may affect its relationship with water density and/or raloxifene [29]. It should be noted that the architecture of cancellous bone introduces more structural variability into the specimens, which may make it more difficult to detect a subtle effect of raloxifene on the mechanical properties of bone.

Our analysis did not demonstrate significant results for screw pull-out, which limits the clinical translation of this experiment. A post hoc power analysis of the two-way analysis of variance indicates power values of 0.288, 0.364, and 0.104 for treatment, screw location, and treatment x screw location, respectively. An important next step in this line of investigation would involve increasing budget and sample size to improve statistical power; however, this may not be practical. Further work should also include testing constructs with more clinical applicability. Rather than using individual screw pull-out testing as a proxy for failure of a fracture fixation construct, an entire locking plate-screw construct could be tested. While significantly more costly, testing a locking plate-screw construct would provide additional clinical relevance to the experiment. Furthermore, a plate-screw construct may make the detection of a smaller clinical effect possible due to the summative effect of increasing pull-out strength of all the screws in the construct. Additionally, in vivo experiments are likely required to determine whether localized application of raloxifene will improve implant fixation. Other research could focus on osteoporotic fracture care that does not involve fixation, such as the prevention of progressive loss of height in vertebral body compression fractures.

This study included several limitations. There were several parameters within this experimental protocol, including type of bone (animal vs. human), bone type (cancellous vs. cortical, mature vs. immature), raloxifene concentration, duration of bone exposure to raloxifene, and conditions of raloxifene treatment (temperature, solution, antibiotics) that remain untested and may confound the effectiveness of raloxifene. Soaking bone in Raloxifene is not feasible clinically and would require a vehicle for delivery, which was not considered in the study. This study would be strengthened by quantification of degree of mineralization, bound water concentration, total water content, and microarchitectural assessment of bones using μCT. Finally, while the variables used in this study did not yield the expected result, there may be other conditions in which bone material properties may be enhanced. If more promising and reproducible protocols are established, larger sample sizes would certainly be justified and provide valuable insight.

Fixation of osteoporotic fractures represents a major clinical challenge. Surgical repair is often not considered as an option due to the high likelihood of failure - even with the most advanced implants and construct designs [38–40]. Current research overwhelmingly focuses on the design of new implants and modification of current implants and construct designs. However, it is important to explore biologic treatments that can address the fundamental, underlying problem of bone quality. This will allow improved outcomes in all aspects of osteoporotic fracture care, from improving surgical outcomes to increasing the number of patients who can be treated with surgery. It is also important to appreciate the context of a biological solution and the potential to be immediately applicable to everything from proximal humerus fractures to osteoporotic vertebral compression fractures to hip fractures.

## Conclusions

This study found that raloxifene immersion did not change the mechanical properties of cancellous bone tissue from fetal bovine specimens and human cadavers. Future work is required to find an operative intervention capable of rapidly improving the mechanical properties of osteoporotic bone in the acute fracture setting. This could significantly decrease the burden of osteoporotic fractures in aging populations around the world.

## Data Availability

The datasets used and/or analyzed during the current study are available from the corresponding author on reasonable request.

## Abbreviations

IACUC: Institutional Animal Care and Use Committee
IRB: Institutional Review Board
DMSO: Dimethylsulfoxide
PBS: Phosphate Buffered Saline
DEXA: Dual energy X-ray absorptiometry

## References

[1] Johnell O, Kanis JA (2006). An estimate of the worldwide prevalence and disability associated with osteoporotic fractures. Osteoporosis Int.

[2] Burge R, Dawson-Hughes B, Solomon DH, Wong JB, King A, Tosteson A (2007). Incidence and economic burden of osteoporosis-related fractures in the United States, 2005–2025. J Bone Miner Res.

[3] Wright NC, Looker AC, Saag KG, Curtis JR, Delzell ES, Randall S, Dawson-Hughes B (2014). The recent prevalence of osteoporosis and low bone mass in the United States based on bone mineral density at the femoral neck or lumbar spine. J Bone Miner Res.

[4] Collard RM, Boter H, Schoevers RA, Oude Voshaar RC (2012). Prevalence of frailty in community-dwelling older persons: a systematic review. J Am Geriatr Soc.

[5] Kim SH, Szabo RM, Marder RA. Epidemiology of humerus fractures in the United States: Nationwide emergency department sample, 2008. Arthritis Care Res. 2012;64(3):–414. 10.1002/acr.21563. 10.1002/acr.21563 22162357

[6] Singer A, Exuzides A, Spangler L, O’Malley C, Colby C, Johnston K, Agodoa I, Baker J, Kagan R (2015). Burden of illness for osteoporotic fractures compared with other serious diseases among postmenopausal women in the United States. Mayo Clin Proc.

[7] Sabesan VJ, Valikodath T, Childs A, Sharma VK (2015). Economic and social impact of upper extremity fragility fractures in elderly patients. Aging Clin Exp Res.

[8] Sproul RC, Iyengar JJ, Devcic Z, Feeley BT (2011). A systematic review of locking plate fixation of proximal humerus fractures. Injury.

[9] Broderick JM, Bruce-Brand R, Stanley E, Mulhall KJ (2013). Osteoporotic Hip fractures: The burden of fixation failure. Sci World J.

[10] Kim WY, Han CH, Park JI, Kim JY. Failure of intertrochanteric fracture fixation with a dynamic hip screw in relation to pre-operative fracture stability and osteoporosis. Int Orthop. 2001. 10.1007/s002640100287. 10.1007/s002640100287 PMC3620783 11820441

[11] Bae JH, Oh JK, Chon CS, Oh CW, Hwang JH, Yoon YC. The biomechanical performance of locking plate fixation with intramedullary fibular strut graft augmentation in the treatment of unstable fractures of the proximal humerus. J Bone Joint Surg Series B. 2011. 10.1302/0301-620X.93B7.26125. 10.1302/0301-620X.93B7.26125 21705567

[12] Cha H, Park KB, Oh S, Jeong J (2017). Treatment of comminuted proximal humeral fractures using locking plate with strut allograft. J Shoulder Elbow Surg.

[13] Hast MW, Chin M, Schmidt EC, Sanville J, Van Osten GK, Mehta S (2020). Mechanical effects of bone substitute and far-cortical locking techniques in 2-part proximal Humerus fracture reconstruction. J Orthop Trauma.

[14] Mattsson P, Alberts A, Dahlberg G, Sohlman M, Hyldahl HC, Larsson S. Resorbable cement for the augmentation of internally-fixed unstable trochanteric fractures. A prospective, randomized multicentre study. J Bone Joint Surg Series B. 2005. 10.1302/0301-620X.87B9.15792. 10.1302/0301-620X.87B9.15792 16129742

[15] Namdari S, Voleti PB, Mehta S (2012). Evaluation of the osteoporotic proximal humeral fracture and strategies for structural augmentation during surgical treatment. J Shoulder Elbow Surg.

[16] Cornell CN (2003). Internal fracture fixation in patients with osteoporosis. J Am Acad Orthop Surg.

[17] Xie Y, Zhang L, Xiong Q, Gao Y, Ge W, Tang P (2019). Bench-to-bedside strategies for osteoporotic fracture: From osteoimmunology to mechanosensation. Bone Res.

[18] Crandall CJ, Newberry SJ, Diamant A, Lim YW, Gellad WF, Booth MJ, Motala A, Shekelle PG (2014). Comparative effectiveness of pharmacologic treatments to prevent fractures: An updated systematic review. Ann Intern Med.

[19] Barrionuevo P, Kapoor E, Asi N, Alahdab F, Mohammed K, Benkhadra K, Almasri J, Farah W, Sarigianni M, Muthusamy K, al Nofal A, Haydour Q, Wang Z, Murad MH (2019). Efficacy of pharmacological therapies for the prevention of fractures in postmenopausal women: A network meta-analysis. J Clin Endocrinol Metab.

[20] Molvik H, Khan W (2015). Bisphosphonates and their influence on fracture healing: a systematic review. Osteoporos Int.

[21] Seebach C, Kurth A, Marzi I (2007). The influence of bisphosphonates on fracture healing. Orthopade.

[22] Hak DJ (2018). The biology of fracture healing in osteoporosis and in the presence of anti-osteoporotic drugs. Injury.

[23] Kates SL, Ackert-Bicknell CL (2016). How do bisphosphonates affect fracture healing?. Injury.

[24] Xue D, Li F, Chen G, Yan S, Pan Z. Do bisphosphonates affect bone healing? A meta-analysis of randomized controlled trials. J Orthop Surg Res. 2014. 10.1186/1749-799X-9-45. 10.1186/1749-799X-9-45 PMC4058448 24902588

[25] Shi Z, Zhou H, Pan B, Lu L, Liu J, Kang Y, Yao X, Feng S (2016). Effectiveness of teriparatide on fracture healing: A systematic review and meta-analysis. Plos One.

[26] Delmas PD, Bjarnason NH, Mitlak BH, et al. Effects of raloxifene on bone mineral density, serum cholesterol concentrations, and uterine endometrium in postmenopausal women. N Engl J Med. 1997. 10.1056/NEJM199712043372301. 10.1056/NEJM199712043372301 9385122

[27] Rey JRC, Cervino EV, Rentero ML, Crespo EC, Álvaro AO, Casillas M. Raloxifene: Mechanism of Action, Effects on Bone Tissue, and Applicability in Clinical Traumatology Practice. Open Orthop J. 2009. 10.2174/1874325000903010014. 10.2174/1874325000903010014 PMC2687107 19516920

[28] Gallant MA, Brown DM, Hammond M, Wallace JM, du J, Deymier-Black AC, Almer JD, Stock SR, Allen MR, Burr DB (2014). Bone cell-independent benefits of raloxifene on the skeleton: A novel mechanism for improving bone material properties. Bone.

[29] Garnero P, Borel O, Gineyts E, Duboeuf F, Solberg H, Bouxsein ML, Christiansen C, Delmas PD (2006). Extracellular post-translational modifications of collagen are major determinants of biomechanical properties of fetal bovine cortical bone. Bone..

[30] Kumar V, Green S, Stack G, Berry M, Jin JR, Chambon P. Functional domains of the human estrogen receptor. Cell. 1987. 10.1016/0092-8674(87)90581-2. 10.1016/0092-8674(87)90581-2 3690665

[31] Cattani-Lorente M, Rizzoli R, Ammann P (2013). In vitro bone exposure to strontium improves bone material level properties. Acta Biomaterialia.

[32] Nyman JS, Roy A, Shen X, Acuna RL, Tyler JH, Wang X. The influence of water removal on the strength and toughness of cortical bone. J Biomech. 2006. 10.1016/j.jbiomech.2005.01.012. 10.1016/j.jbiomech.2005.01.012 PMC1941695 16488231

[33] Krege JB, Aref MW, McNerny E, Wallace JM, Organ JM, Allen MR (2016). Reference point indentation is insufficient for detecting alterations in traditional mechanical properties of bone under common experimental conditions. Bone..

[34] Currey JD (1988). The effect of porosity and mineral content on the Young’s modulus of elasticity of compact bone. J Biomech.

[35] Zioupos P, Currey JD, Hamer AJ (1999). The role of collagen in the declining mechanical properties of aging human cortical bone. J Biomed Mater Res.

[36] Wang X, Shen X, Li X, Mauli AC (2002). Age-related changes in the collagen network and toughness of bone. Bone..

[37] Broz JJ, Simske SJ, Greenberg AR (1995). Material and compositional properties of selectively demineralized cortical bone. J Biomech.

[38] Von Rüden C, Augat P (2016). Failure of fracture fixation in osteoporotic bone. Injury.

[39] Marongiu G, Mastio M, Capone A (2013). Current options to surgical treatment in osteoporotic fractures. Aging Clinical and Experimental Research.

[40] Konstantinidis L, Helwig P, Hirschmüller A, Langenmair E, Südkamp NP, Augat P (2016). When is the stability of a fracture fixation limited by osteoporotic bone?. Injury.

